# Supplementary material for: Aberrant (pro)renin receptor expression induces genomic instability in pancreatic ductal adenocarcinoma through upregulation of SMARCA5/SNF2H
Source: Commun Biol. 2020 Nov 27;3:724. doi: 10.1038/s42003-020-01434-x (PMC7695732; doi:10.1038/s42003-020-01434-x)

Supplementary information

Aberrant (pro)renin receptor expression induces genomic instability in pancreatic ductal adenocarcinoma through upregulation of SMARCA5/SNF2H

Yuki Shibayama, Kazuo Takahashi, Hisateru Yamaguchi, Jun Yasuda, Daisuke Yamazaki, Asadur Rahman, Takayuki Fujimori, Yoshihide Fujisawa, Shinji Takai, Toru Furukawa, Tsutomu Nakagawa, Hiroyuki Ohsaki, Hideki Kobara, Jing Hao Wong, Tsutomu Masaki, Yukio Yuzawa, Hideyasu Kiyomoto, Shinichi Yachida, Akihiro Fujimoto, Akira Nishiyama

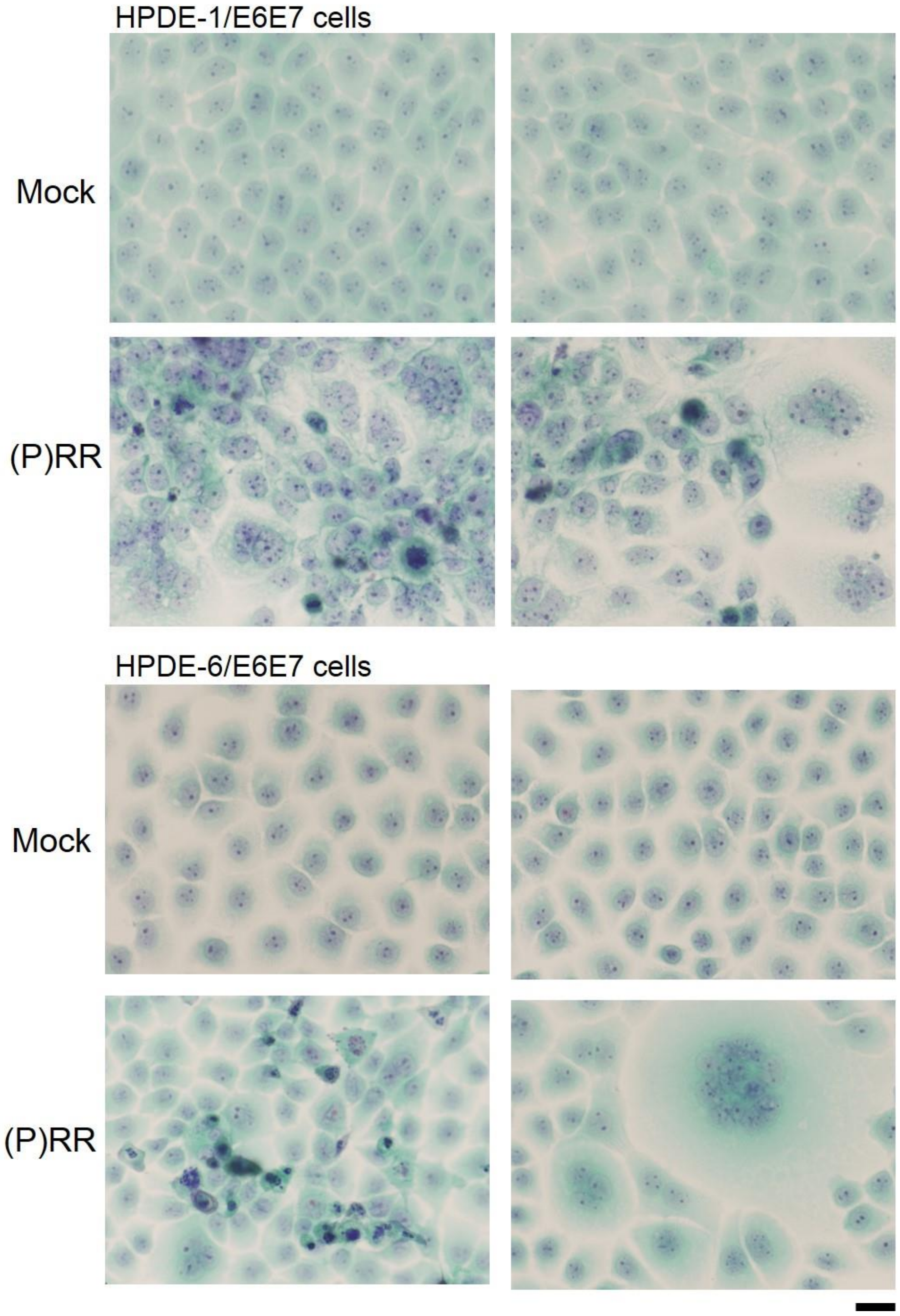

Supplementary Figure 1. (P)RR overexpression generates diverse atypical nuclei in HPDE-1/E6E7 and HPDE-6/E6E7 cell population. Papanicolaou stain (×400).

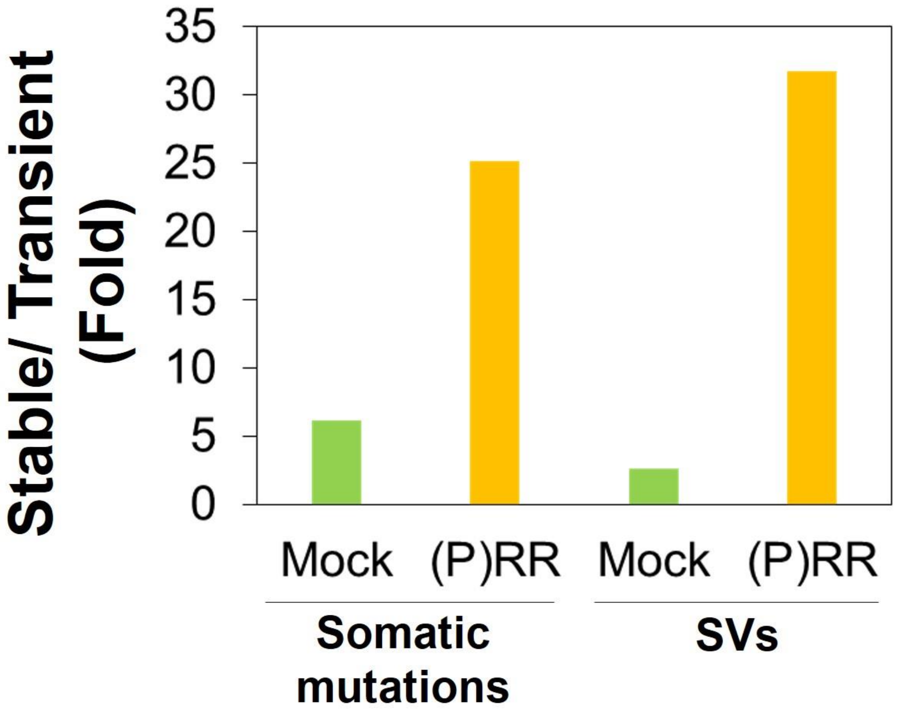

Supplementary Figure 2. Difference of genomic instability between stable Mock expression against transient Mock and stable (P)RR expression against transient (P)RR in HPDE-1/E6E7 cell population.

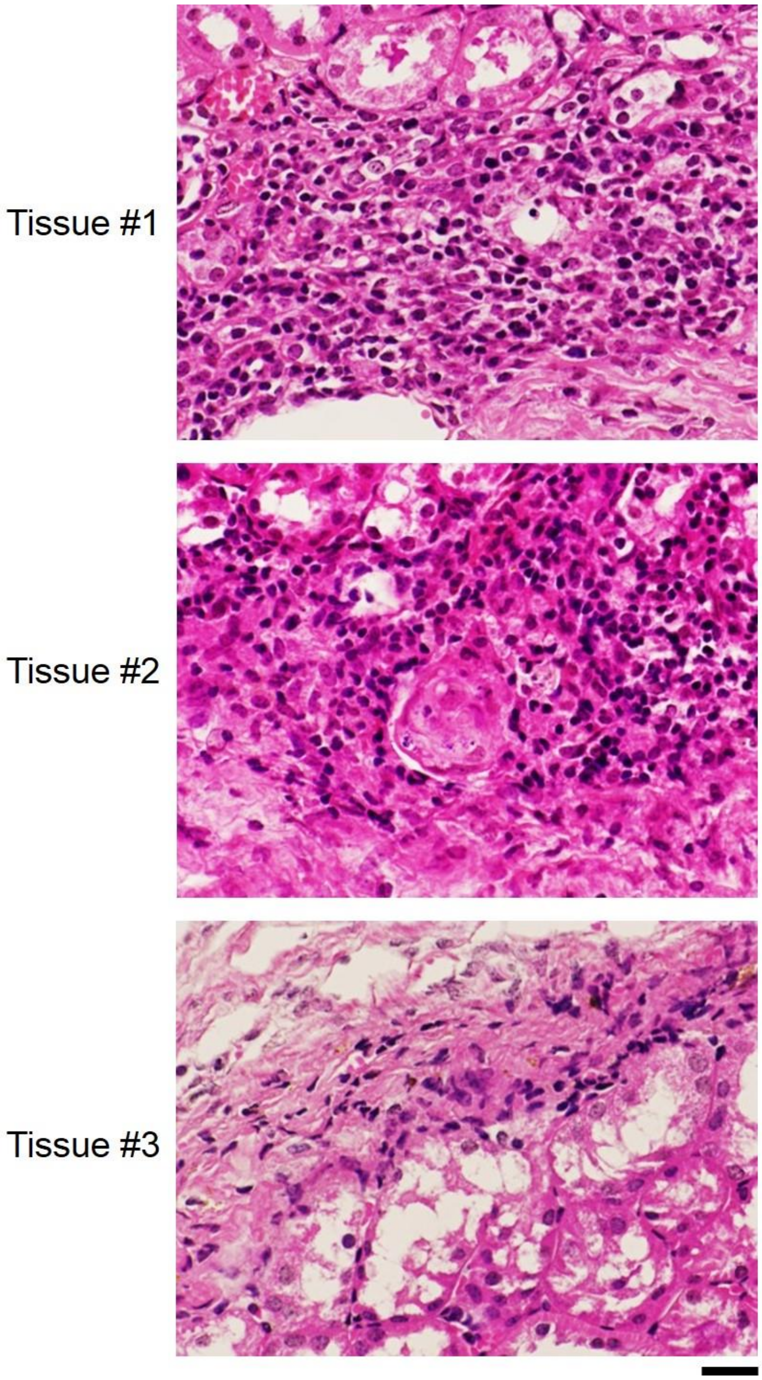

Supplementary Figure 3. (P)RR overexpression generates diverse atypical nuclei. HE stain in each tissue formed by (P)RR-expressing HPDE-1/E6E7 cell population (×400).

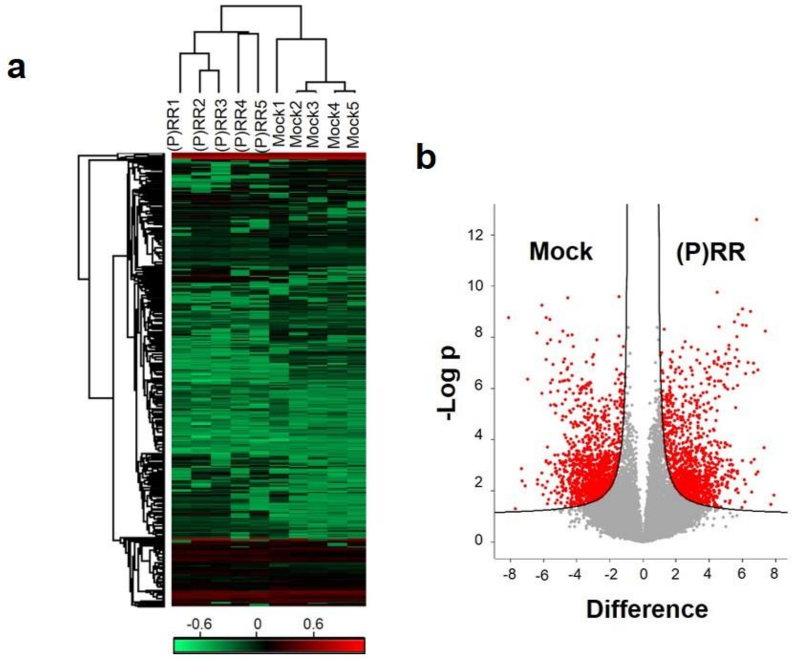

c

Diseases	p-value range	Molecules
Infectious diseases	3.46E-03 - 6.50E-12	31
Organismal Injury and Abnormalities	5.16E-03 - 1.41E-09	72
Developmental Disorder	5.00E-03 - 2.16E-07	29
Hereditary Disorder	3.46E-03 - 2.16E-07	18
Neurological Disease	3.46E-03 - 2.16E-07	17

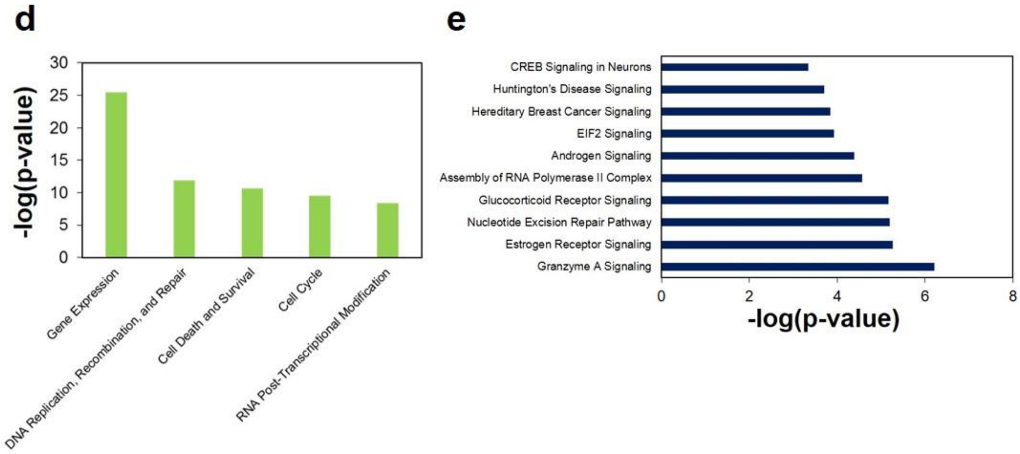

Supplementary Figure 4. **LC-MS/MS analyses. a**, Heat map of label-free quantification (LFQ) areas. **b**, Volcano plots show the p-values *vs*. the difference in peptide abundance in (P)RR-compared with Mock-expressing HPDE-1/E6E7 cells. Red colour shows significantly identified peptides. The level of significant cut-off value is determined based on false discovery rate (FDR) = 0.05 and minimal fold change (S0) = 0.5. **c**, Diseases expected from upregulated molecules under (P)RR overexpression. **d**, Upregulated molecular functions. **e**, Upregulated molecular pathways.

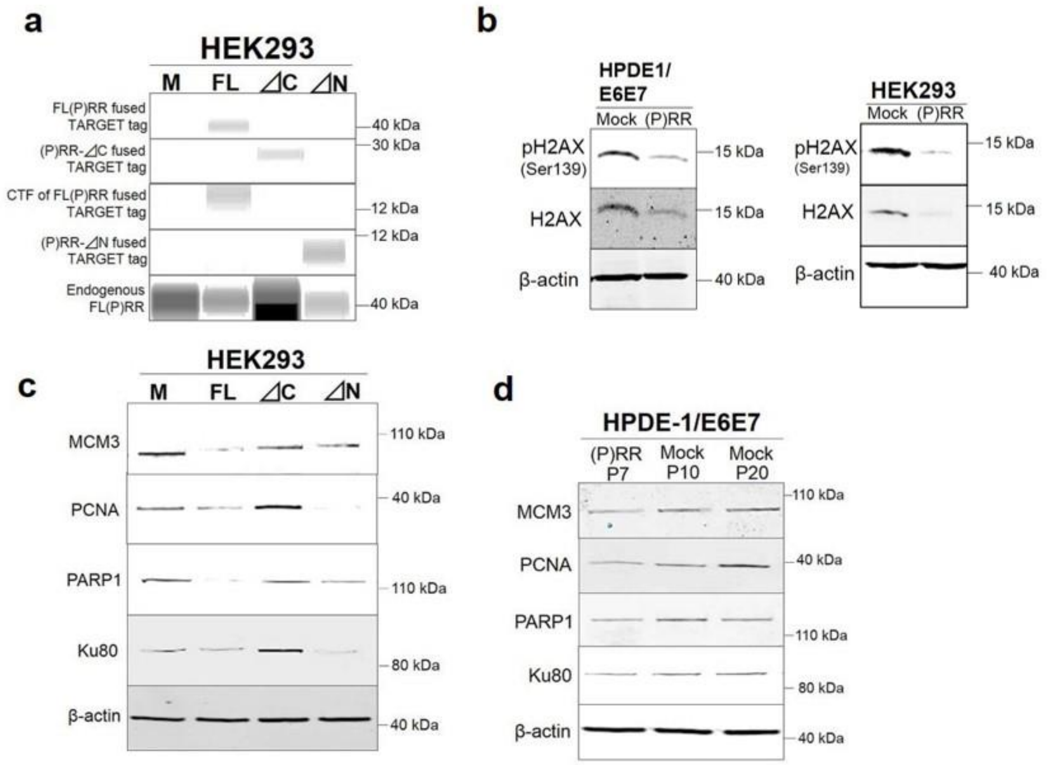

Supplementary Figure 5. **Aberrant (P)RR expression inactivates DNA damage response and downregulates the expression of molecules involved in genomic stability pathways. a**, Confirmation of gene transfection in the vectors with each of Mock (**M**), FL(P)RR (**FL**), NTF of (P)RR (**⊿C**) and CTF of (P)RR (**⊿N**) in HEK293 cells. Endogenous (P)RR was used as a loading control. **b**, DNA damage response in HPDE-1/E6E7 and HEK293 cells with (P)RR overexpression. **c**, Expression of molecules involved in genomic stability pathways in HEK293 cells with the deletion of each domain of (P)RR. **d**, Expression of molecules involved in genomic stability pathways in HPDE-1/E6E7 cells expressing either Mock or (P)RR at different passages.

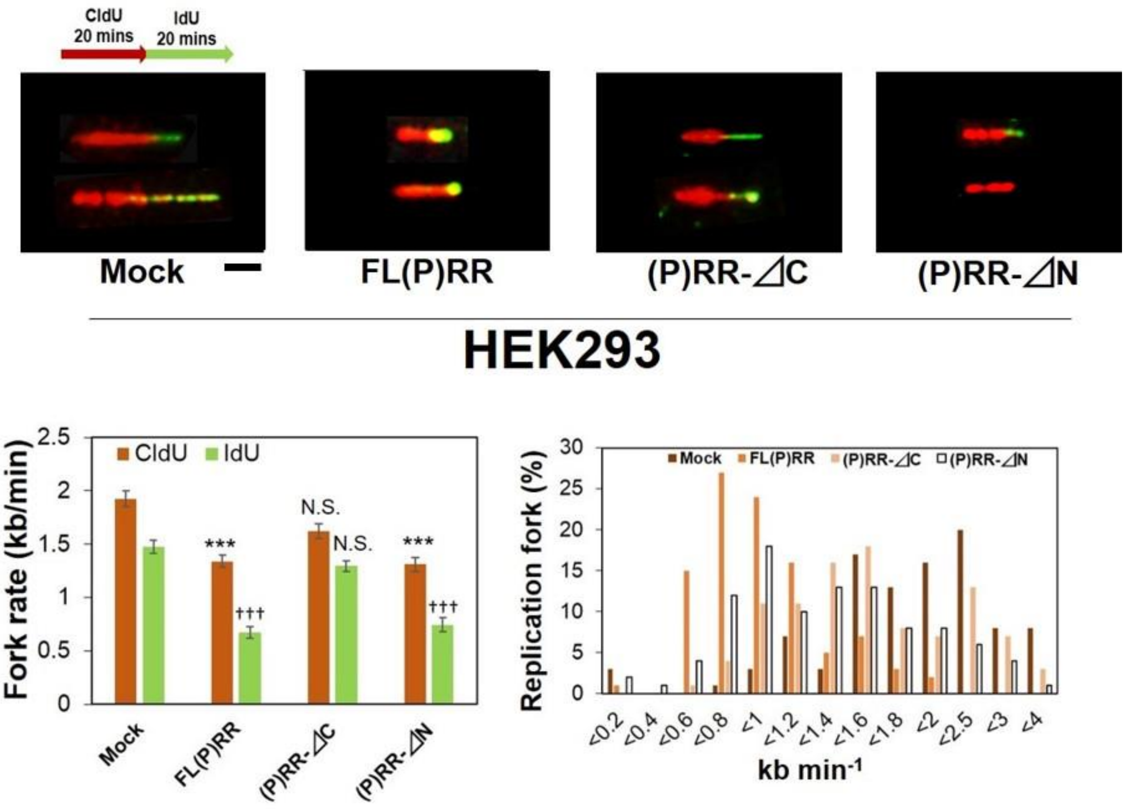

Cells	Mock	FL(P)RR	(P)RR-⊿C	(P)RR-⊿N
Stalled forks (%)	1	22	0	27

Supplementary Figure 6. **CTF of (P)RR induces DNA replication stress in HEK293 cells. a**, Cells were incubated sequentially with 5-chlorodeoxyuridine (CldU) and 5-iododeoxyuridine (IdU) for 20 min each. **Upper**: Representative images of DNA replication fork in cells (×500). **Middle left**: Quantitative evaluation of replication fork rates in cells (mean ± SEM, N = 100 for each, ***P<0.0001 *vs*. Mock in CldU, N.S., not significant, †††P<0.0001 *vs*. Mock in IdU). **Middle right**: Distribution of replication fork rate with CldU in cells. **Lower**: The percentage of stalled forks in cells with the deletion of each domain of (P)RR.

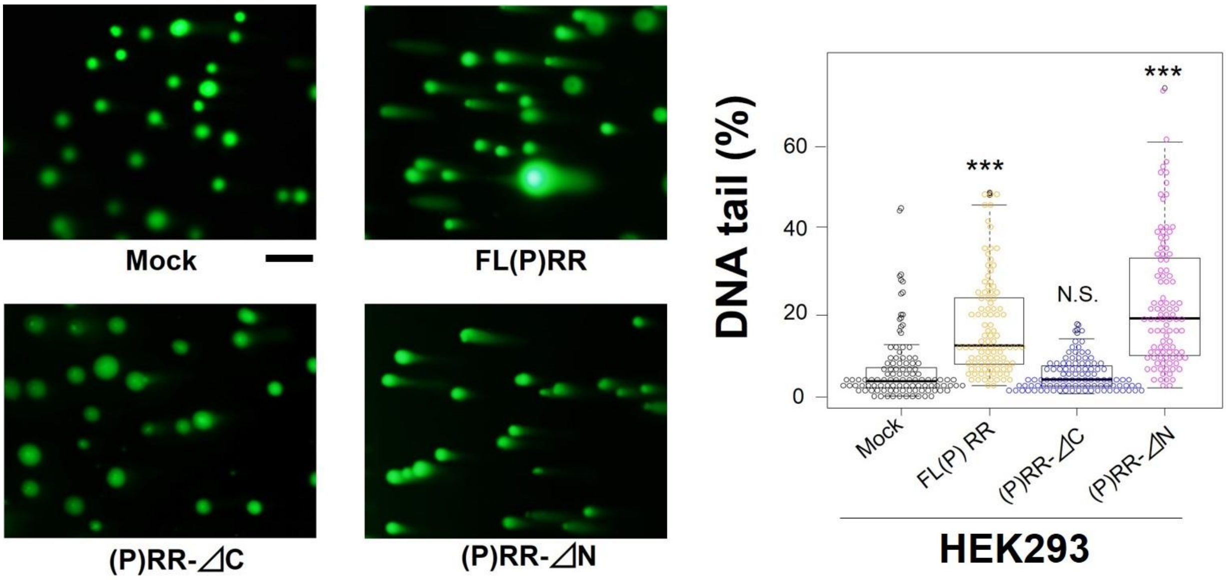

Supplementary Figure 7. **CTF of (P)RR induces the defects of DNA repair capacity**. **Left**: Representative images of single-cell gel electrophoresis in HEK293 cells transfected with the deletion of each domain of (P)RR at three-passage (×100). **Right**: Quantification of the cells with DNA tails (N = 100 for each, ***P<0.001, N.S., not significant). The horizontal line inside the box plot is the median and the vertical lines protruding the box extend to the minimum and the maximum values, respectively. The vertical width of the central box shows the inter-quartile deviation.

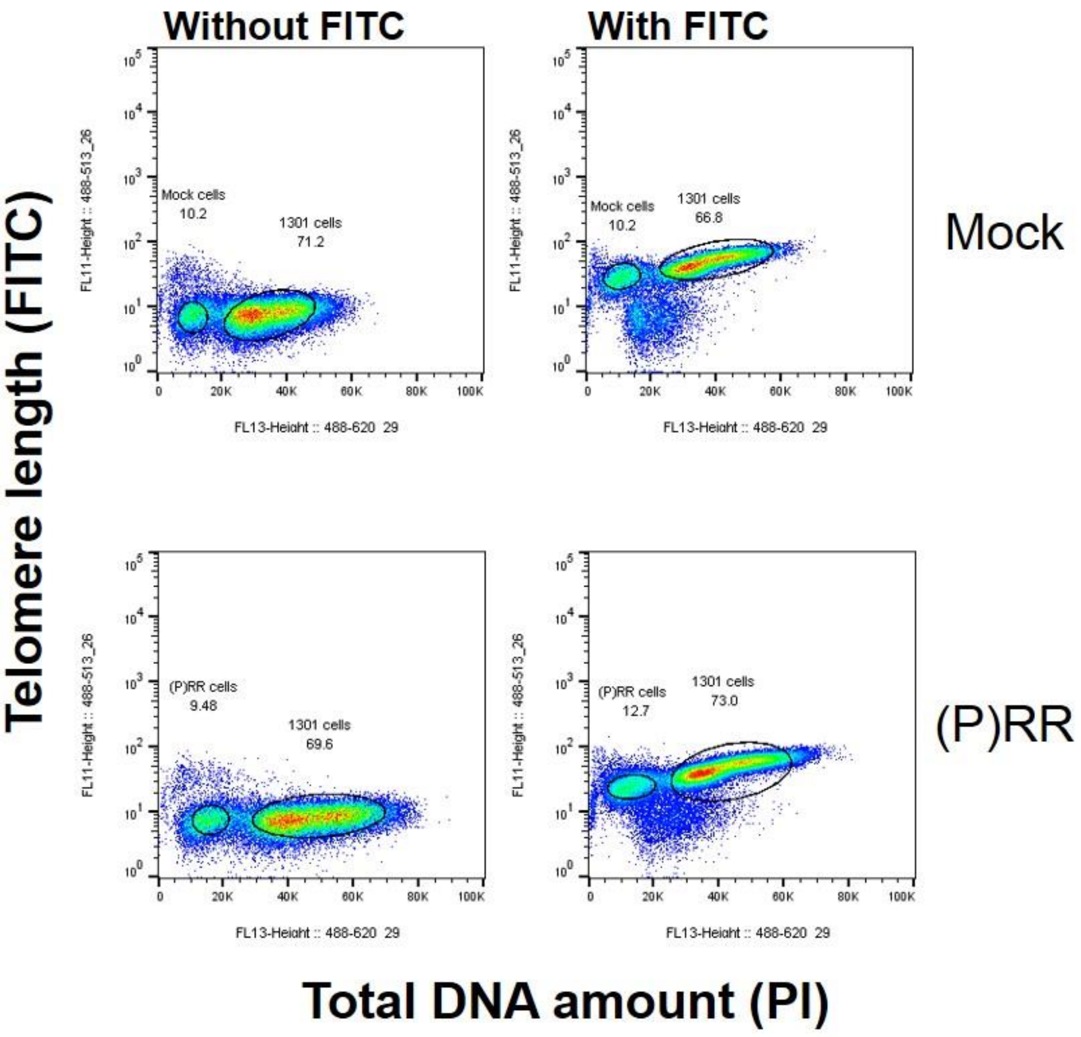

Supplementary Figure 8. **Gating strategy for Flow-FISH in either Mock- or (P)RR-overexpressing HPDE-1/E6E7 cell population.** Numerals show the percentage.

a *Network-1*

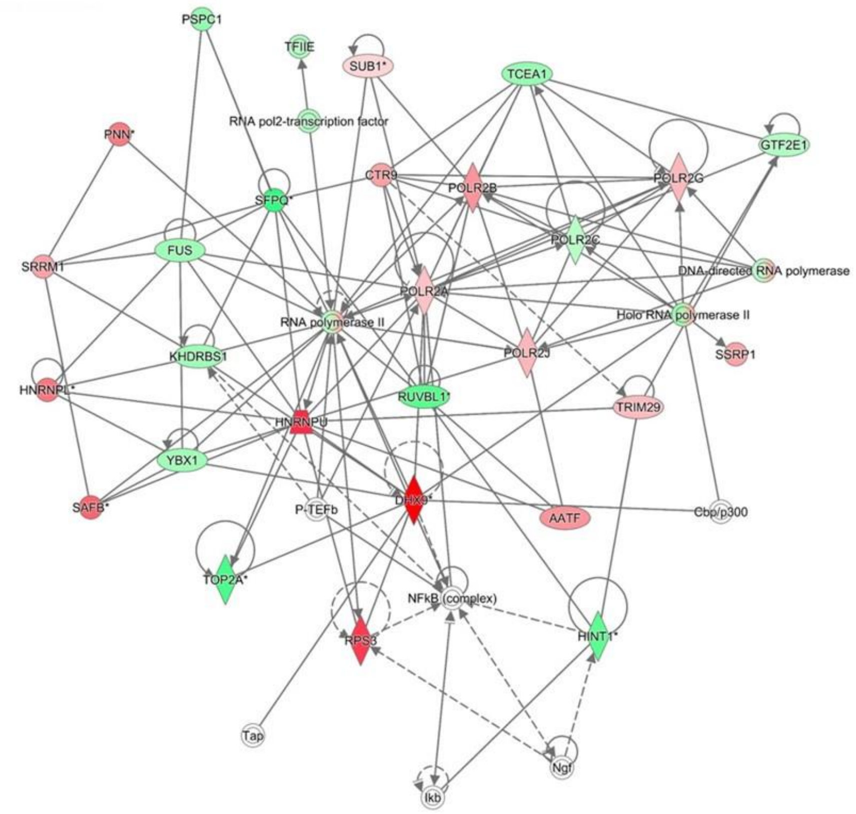

Supplementary Figure 9. **Molecular network affected by (P)RR overexpression in HPDE-1/E6E7 cells. a**, Network-1. Red colour: upregulated molecules; Green colour: downregulated molecules. Straight line: direct interaction. Dotted line: indirect interaction. See the detailed information in Supplementary Data 4.

b *Network-2*

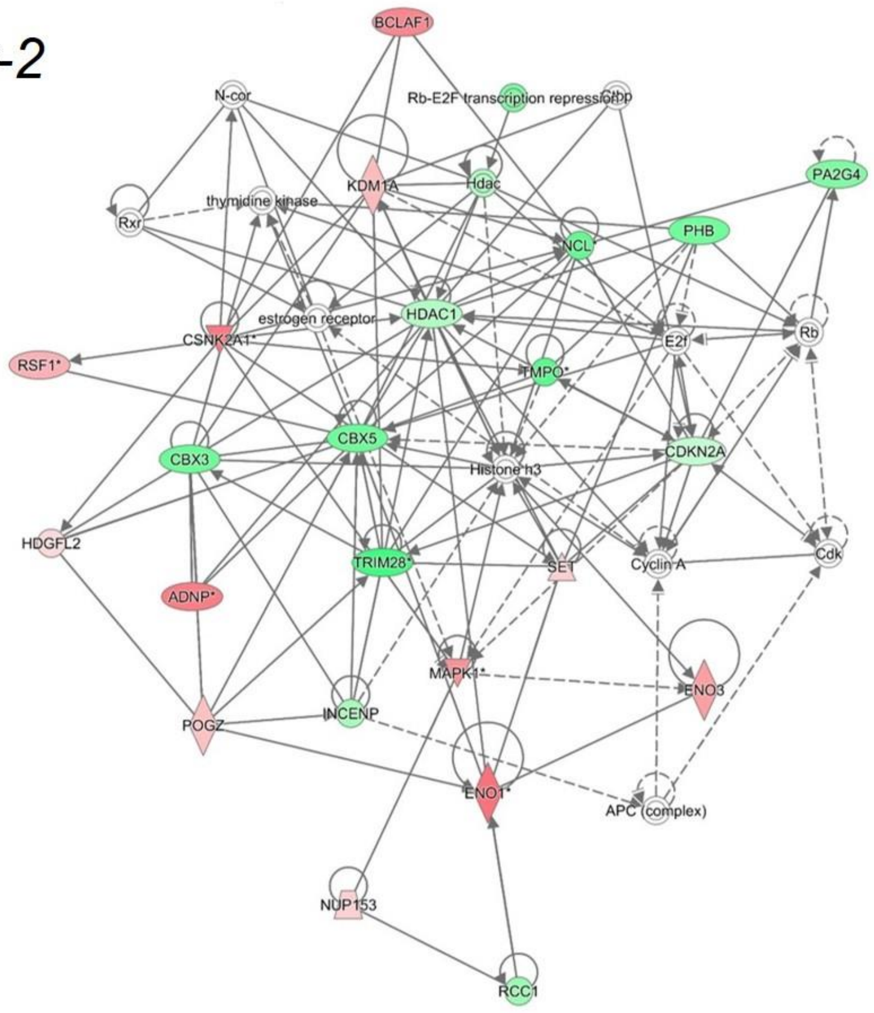

Supplementary Figure 9 (Continued). **Molecular network affected by (P)RR overexpression in HPDE-1/E6E7 cells. b**, Network-2. Red colour: upregulated molecules; Green colour: downregulated molecules. Straight line: direct interaction. Dotted line: indirect interaction. See the detailed information in Supplementary Data 4.

c *Network-4*

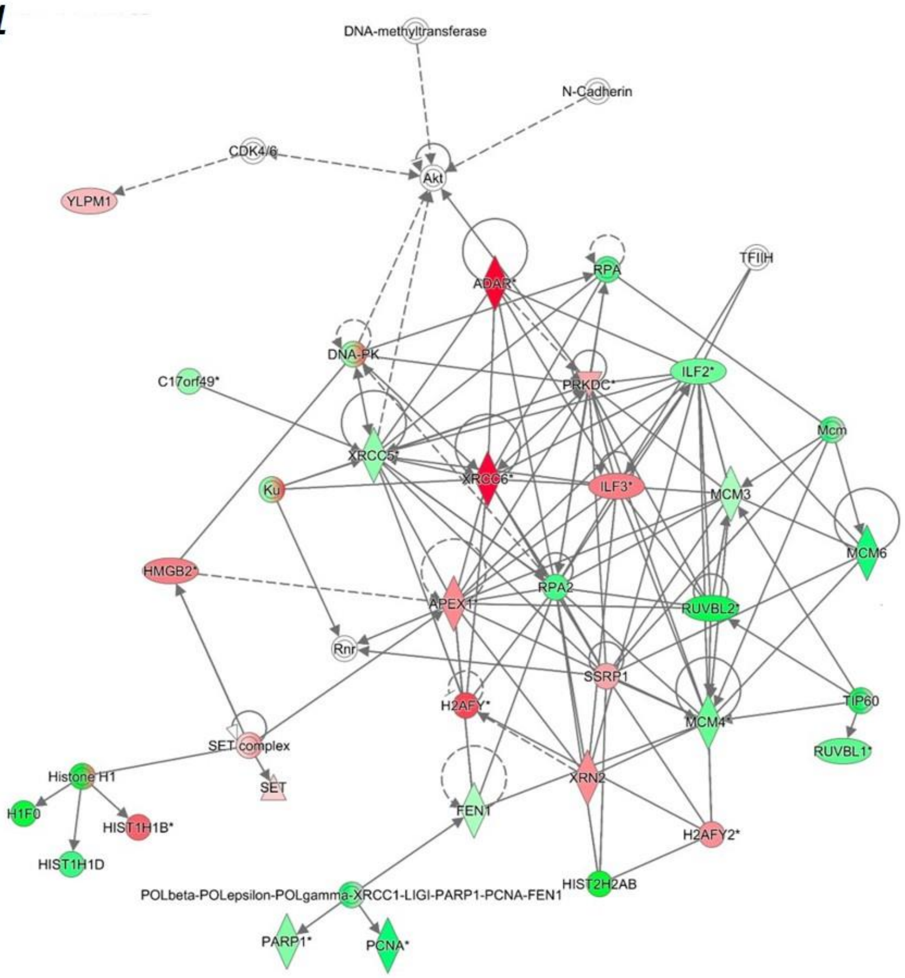

Supplementary Figure 9 (Continued). **Molecular network affected by (P)RR overexpression in HPDE-1/E6E7 cells. c**, Network-4. Red colour: upregulated molecules; Green colour: downregulated molecules. Straight line: direct interaction. Dotted line: indirect interaction. See the detailed information in Supplementary Data 4.

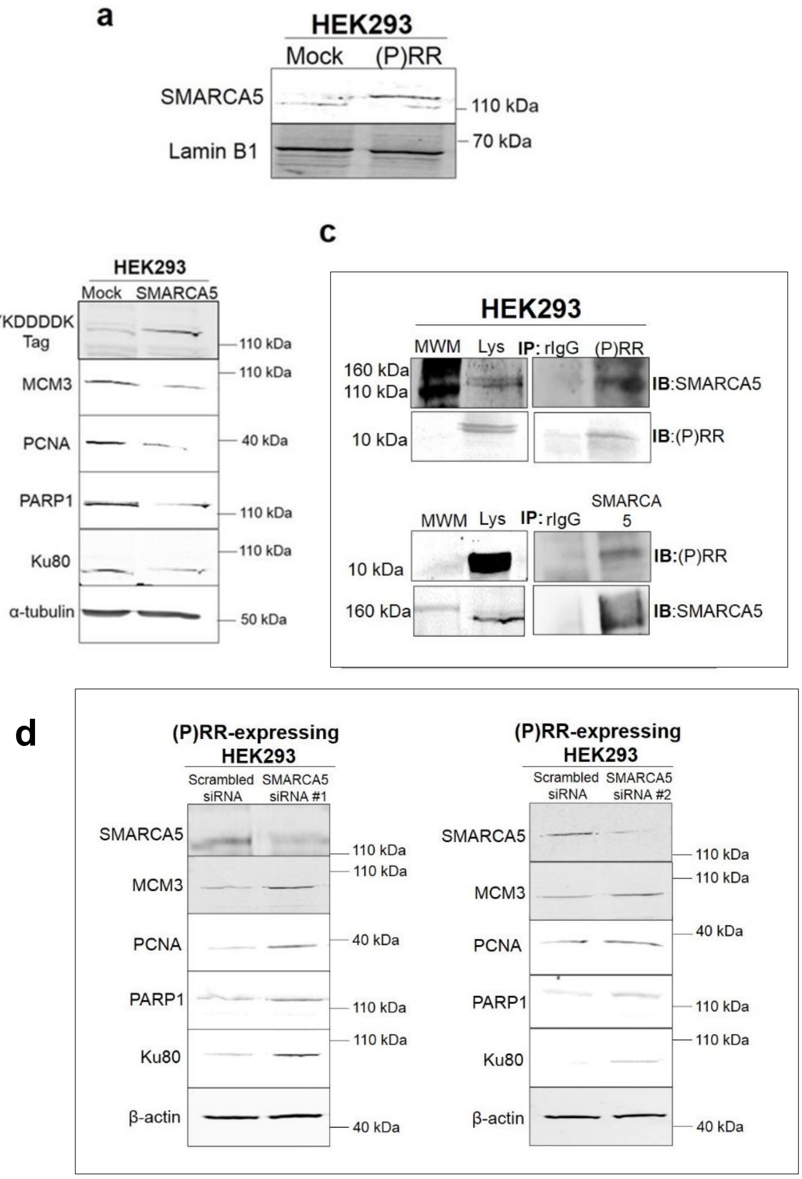

Supplementary Figure 10. (P)RR undergoes a direct molecular interaction with SMARCA5 in HEK293 cells. **a**, SMARCA5 expression in cells with (P)RR overexpression. **b**, Expression of the components responsible for genomic stability pathways in cells with aberrant SMARCA5 expression. **c**, Coimmunoprecipitation between (P)RR and SMARCA5. **MWM**: Molecular Weight Marker; **Lys**: Lysates; **rIgG**: rabbit IgG; **IP**: Immunoprecipitation; **IB**: Immunoblot. **d**, Expression of the components responsible for genomic stability pathways in (P)RR-expressing cells transfected with two different SMARCA5 siRNAs. Consistent results are obtained in three independent experiments for all the Western blot and coimmunoprecipitation.

Unprocessed gel

Fig.1a HPDE-1/E6E7 HPDE-6/E6E7

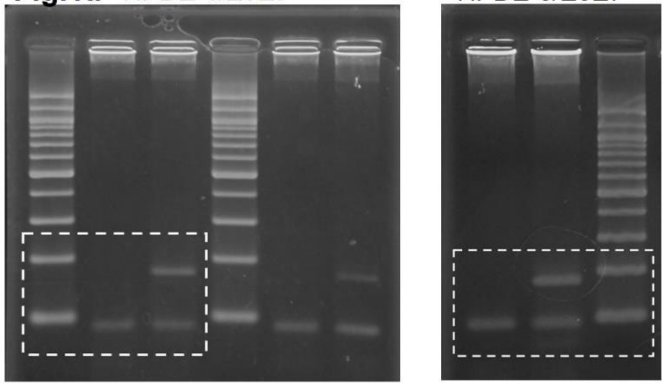

Fig. 2a HPDE-1/E6E7

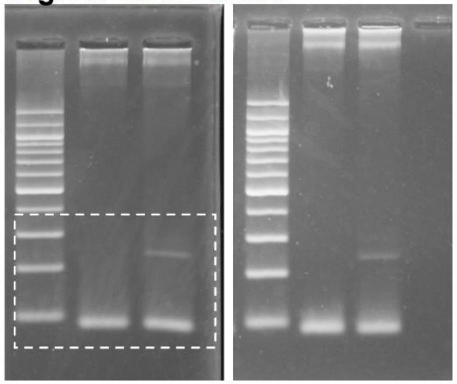

Fig.3a HPDE-1/E6E7

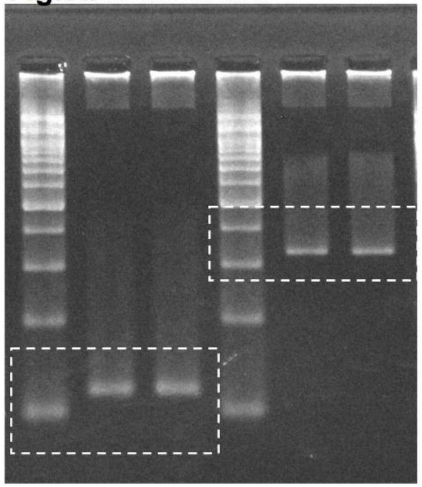

KRAS *CDKN2A*

Dotted box : Processed area

Fig. 4c

Unprocessed blot-1

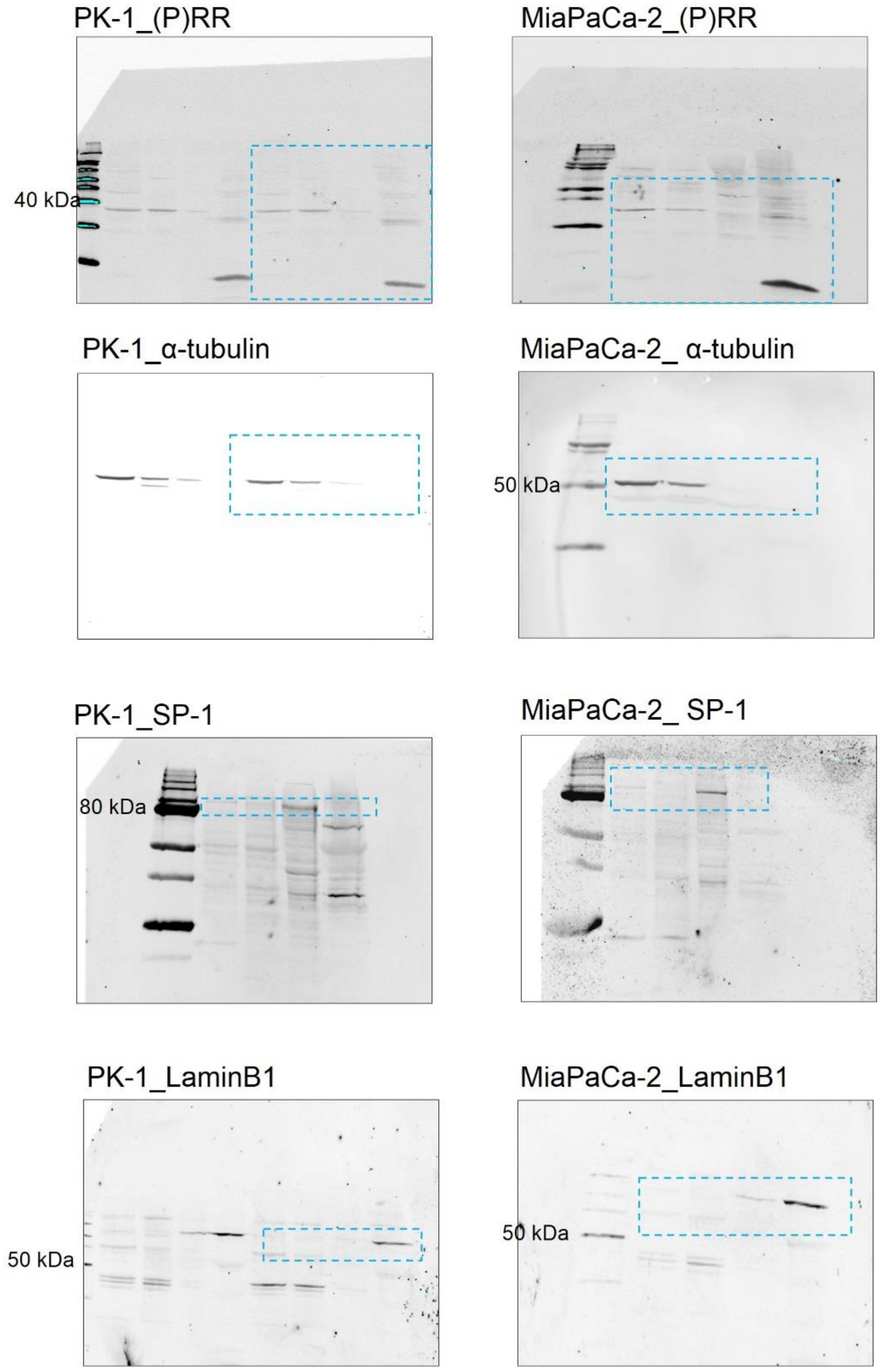

Fig. 5e and Supplementary Fig. 5a

Unprocessed blot-2

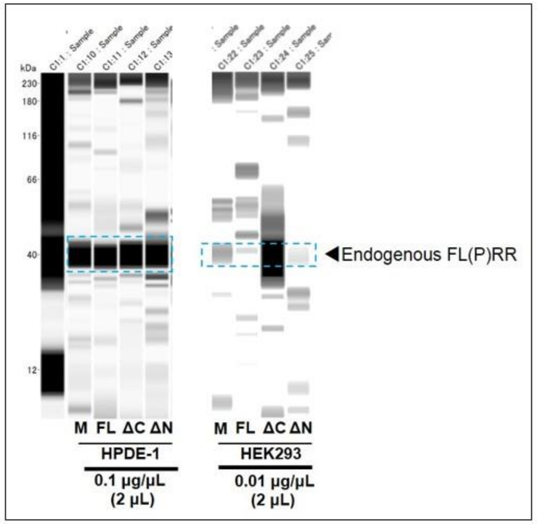

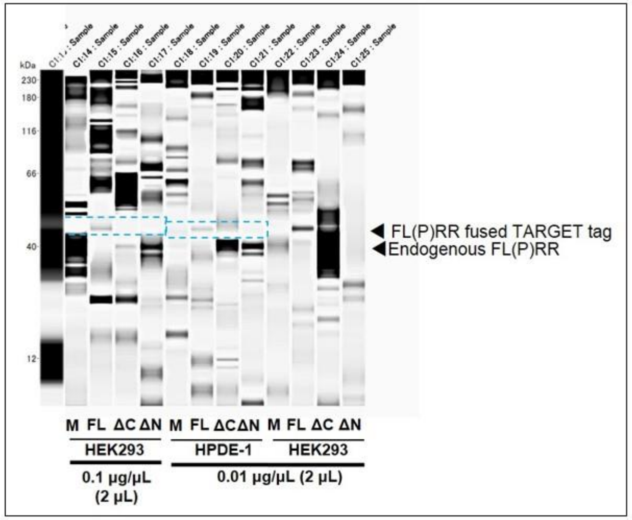

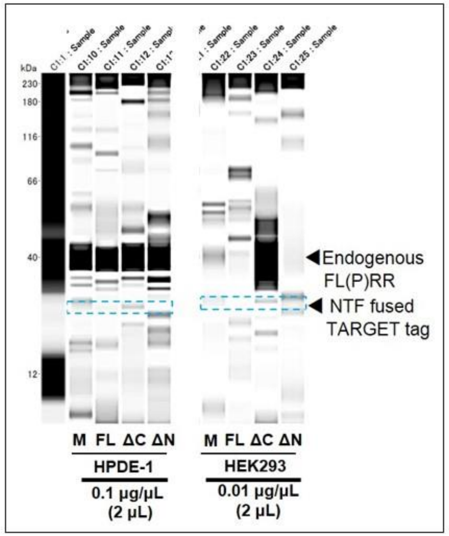

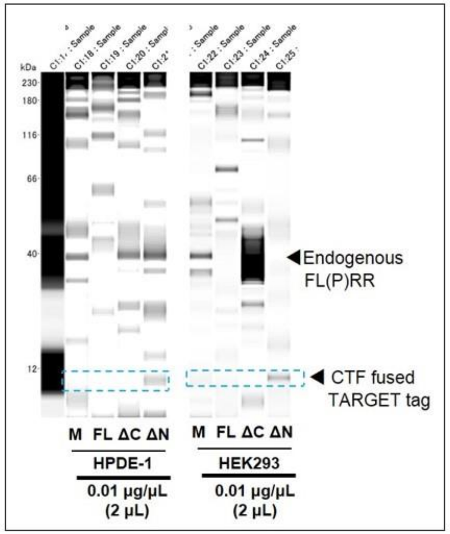

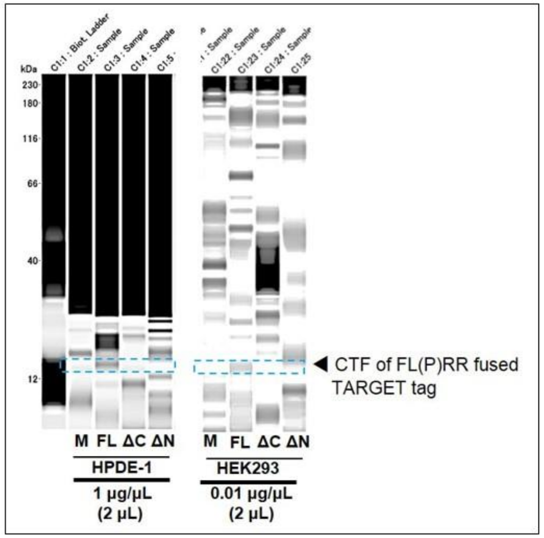

Unprocessed blot-3

Fig. 5f

HPDE-1_MCM3

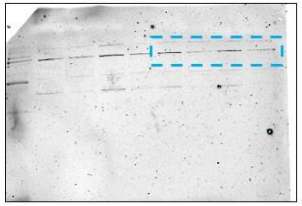

HPDE-1_β-actin

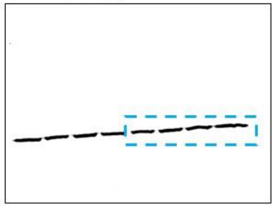

HPDE-1_PCNA

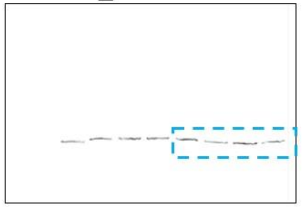

HPDE-1_PARP1

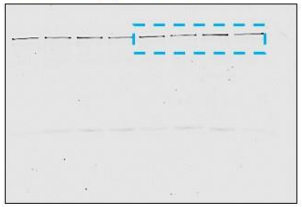

HPDE-1_Ku80

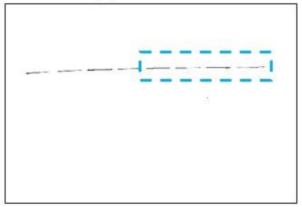

Supplementary Fig. 5c

HEK293_MCM3

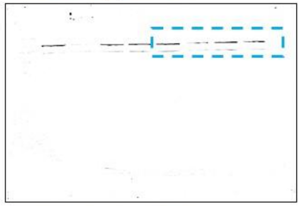

HEK293_β-actin

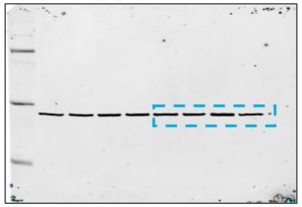

HEK293_PCNA

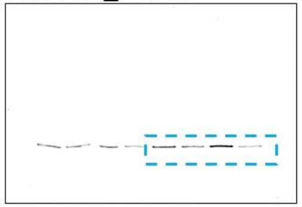

HEK293_PARP1

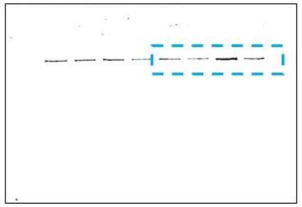

HEK293_Ku80

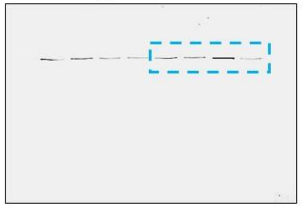

Fig. 5g

Unprocessed blot- 4

HPDE-1_active β-catenin

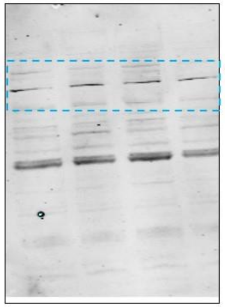

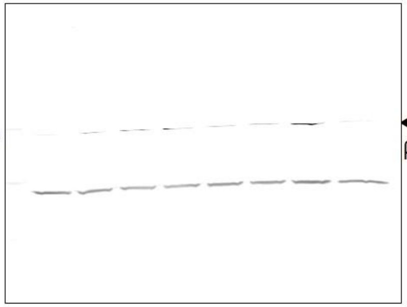

HPDE-1_β-actin

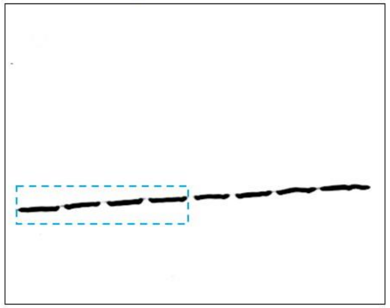

HPDE-1_pLRP6 and LRP6

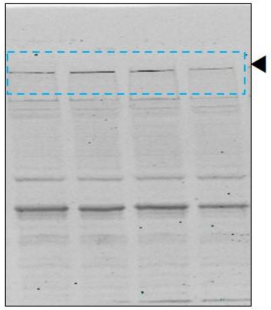

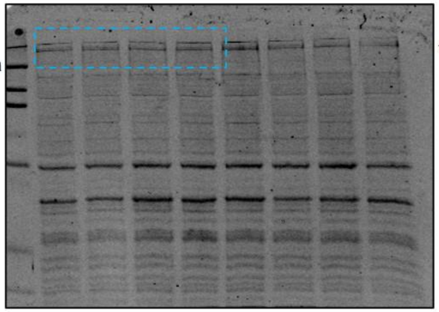

Unprocessed blot-5

Fig. 6c

HPDE-1_SMARCA5

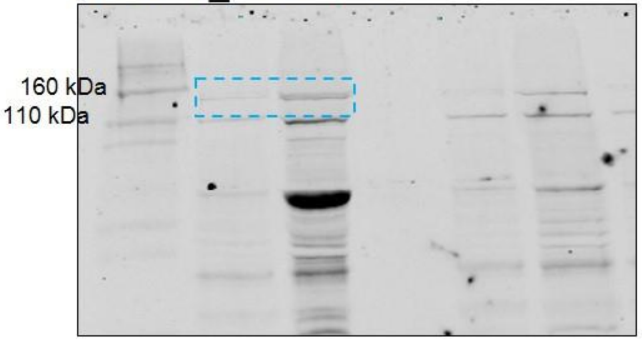

HPDE-1_Lamin B1

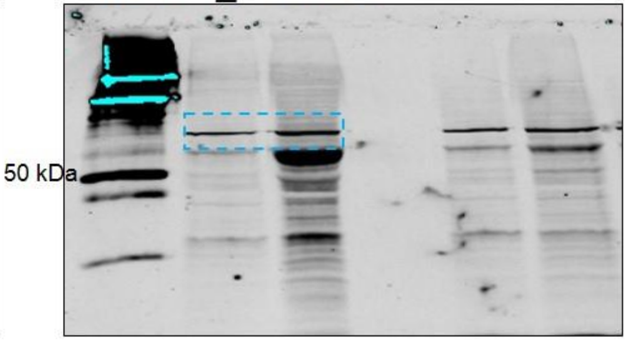

Supplementary Fig. 10a

HEK293_SMARCA5

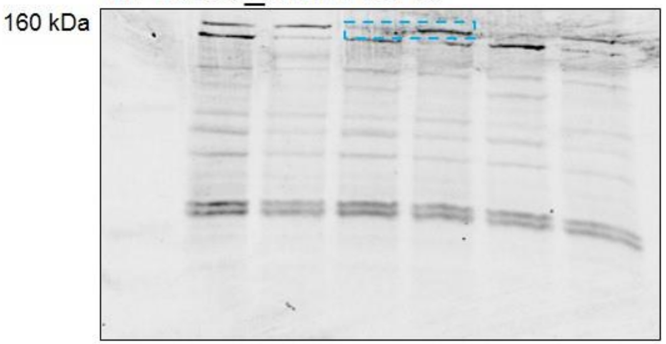

HEK293_Lamin B1

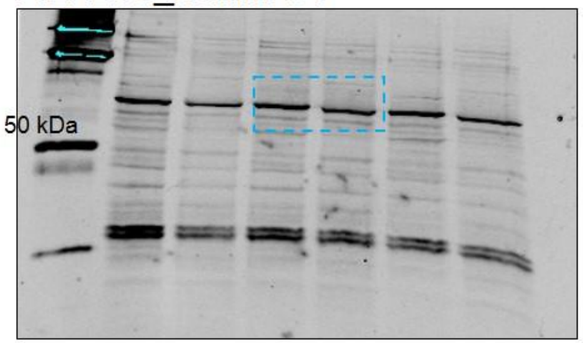

Unprocessed blot- 6

Fig. 6d

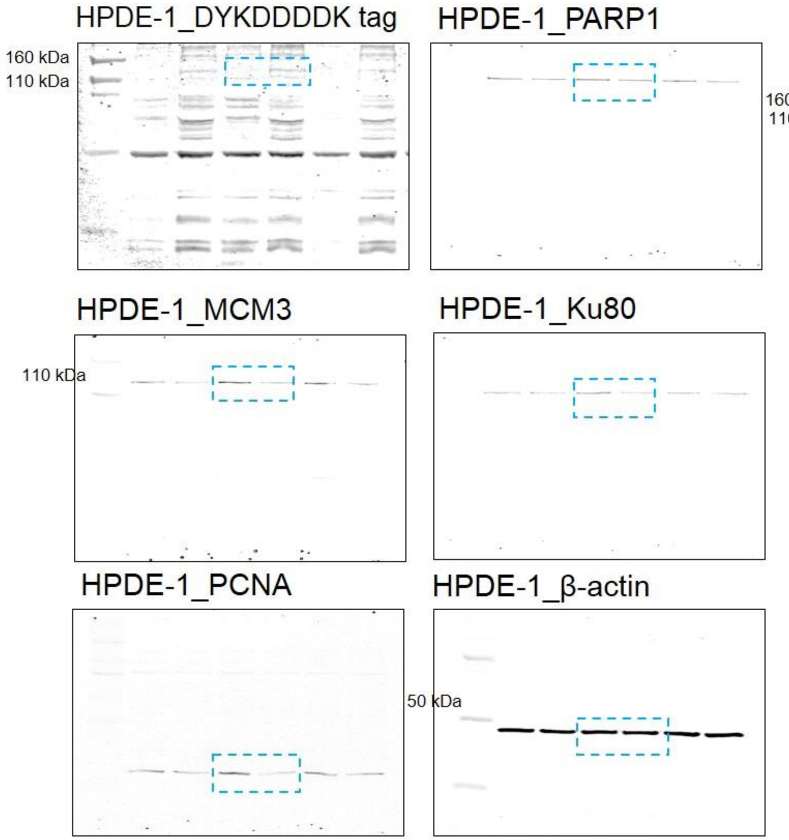

Supplementary Fig.10b

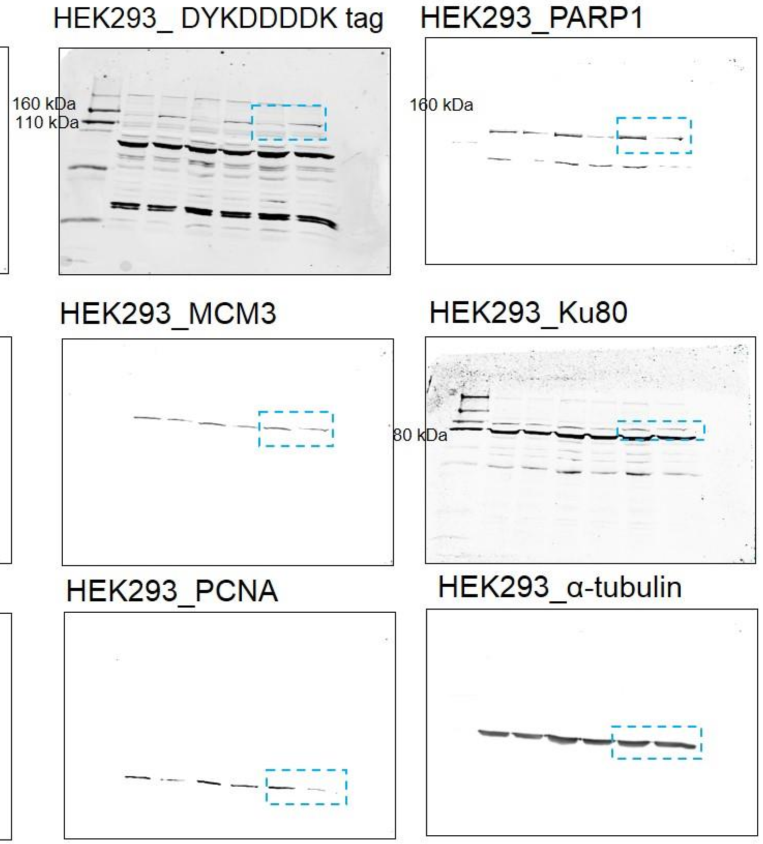

Unprocessed blot-7

Fig. 6e

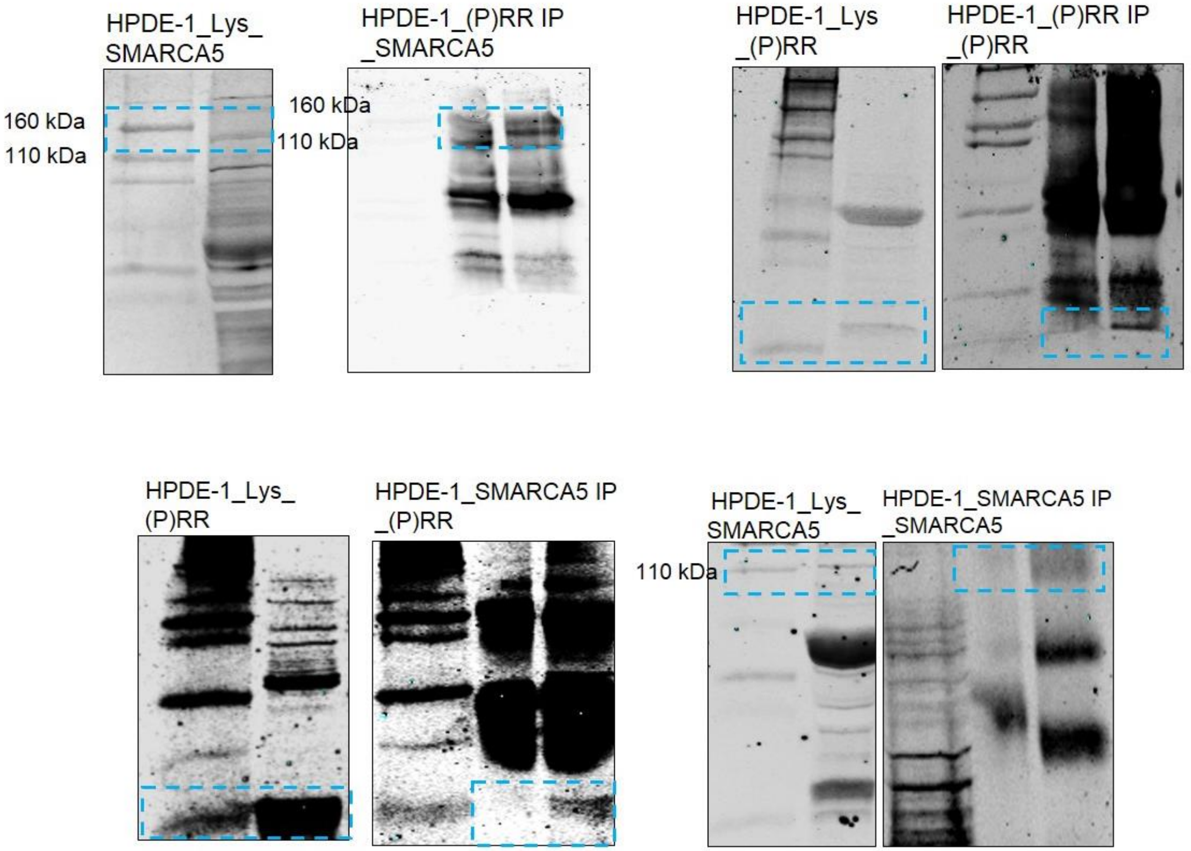

Supplementary Fig. 10c

Unprocessed blot-7

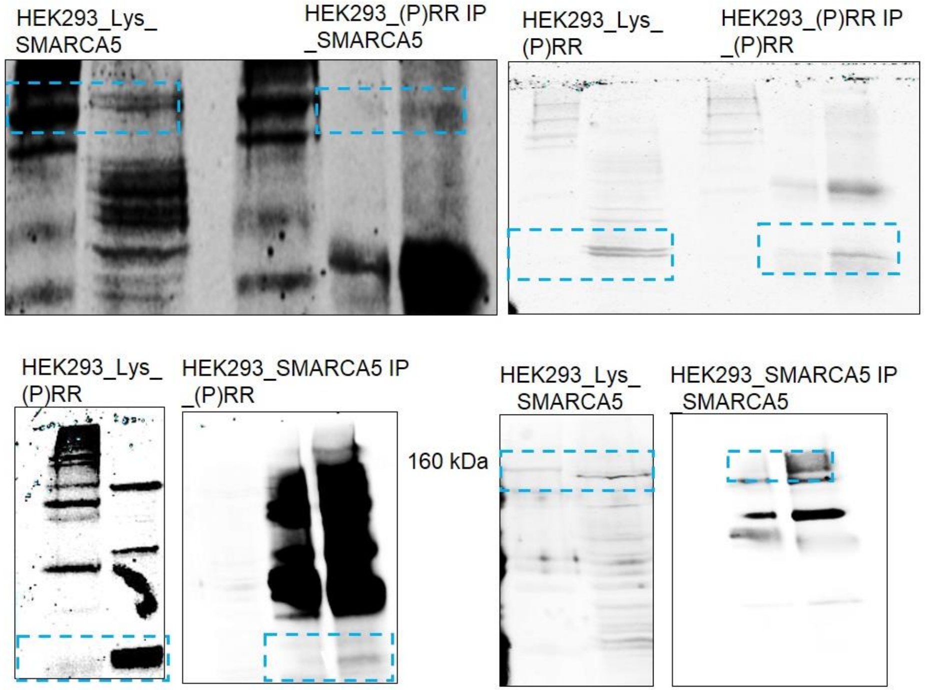

Fig. 6f

Unprocessed blot-8

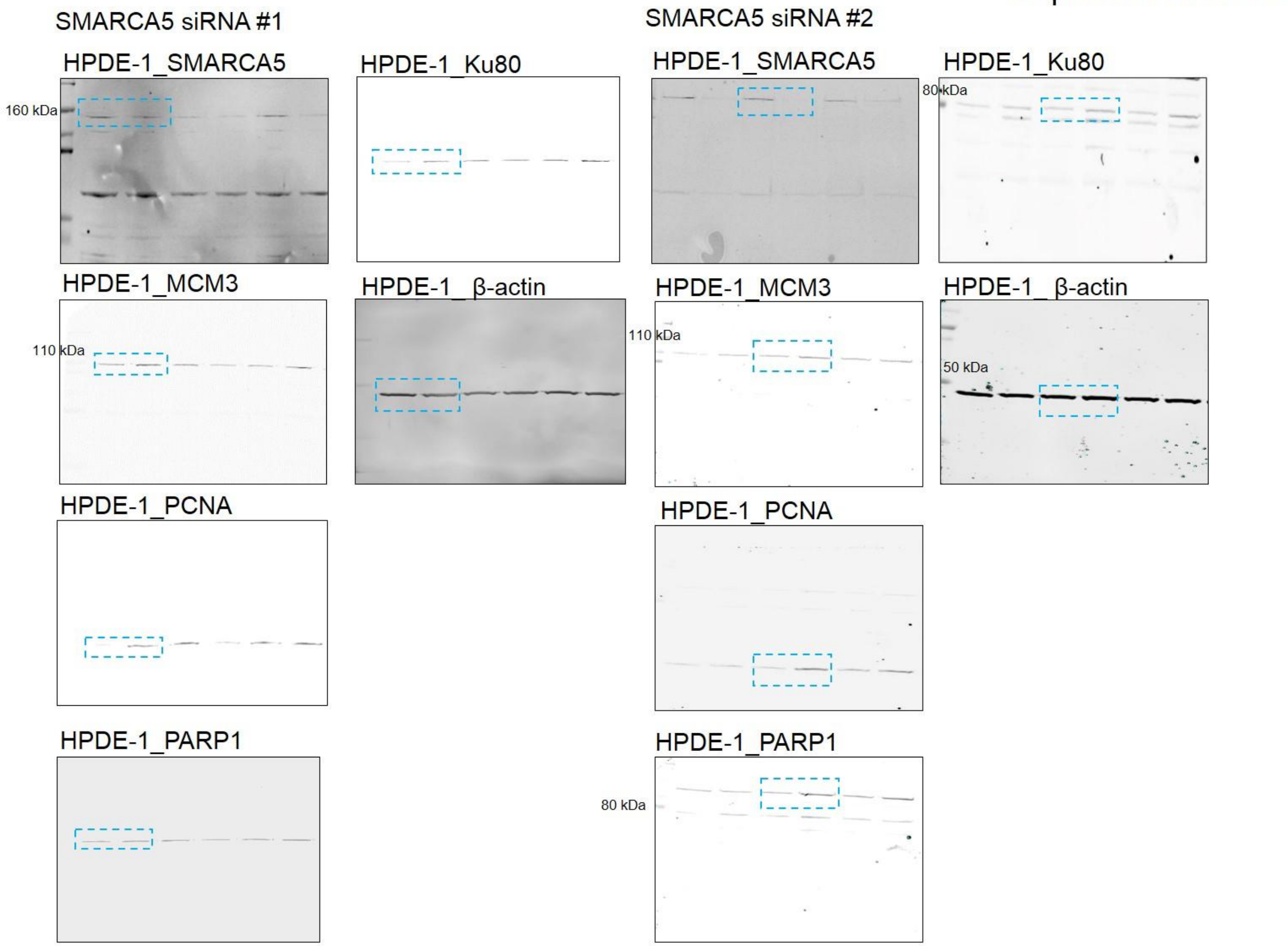

Supplementary Fig. 10d

Unprocessed blot-8

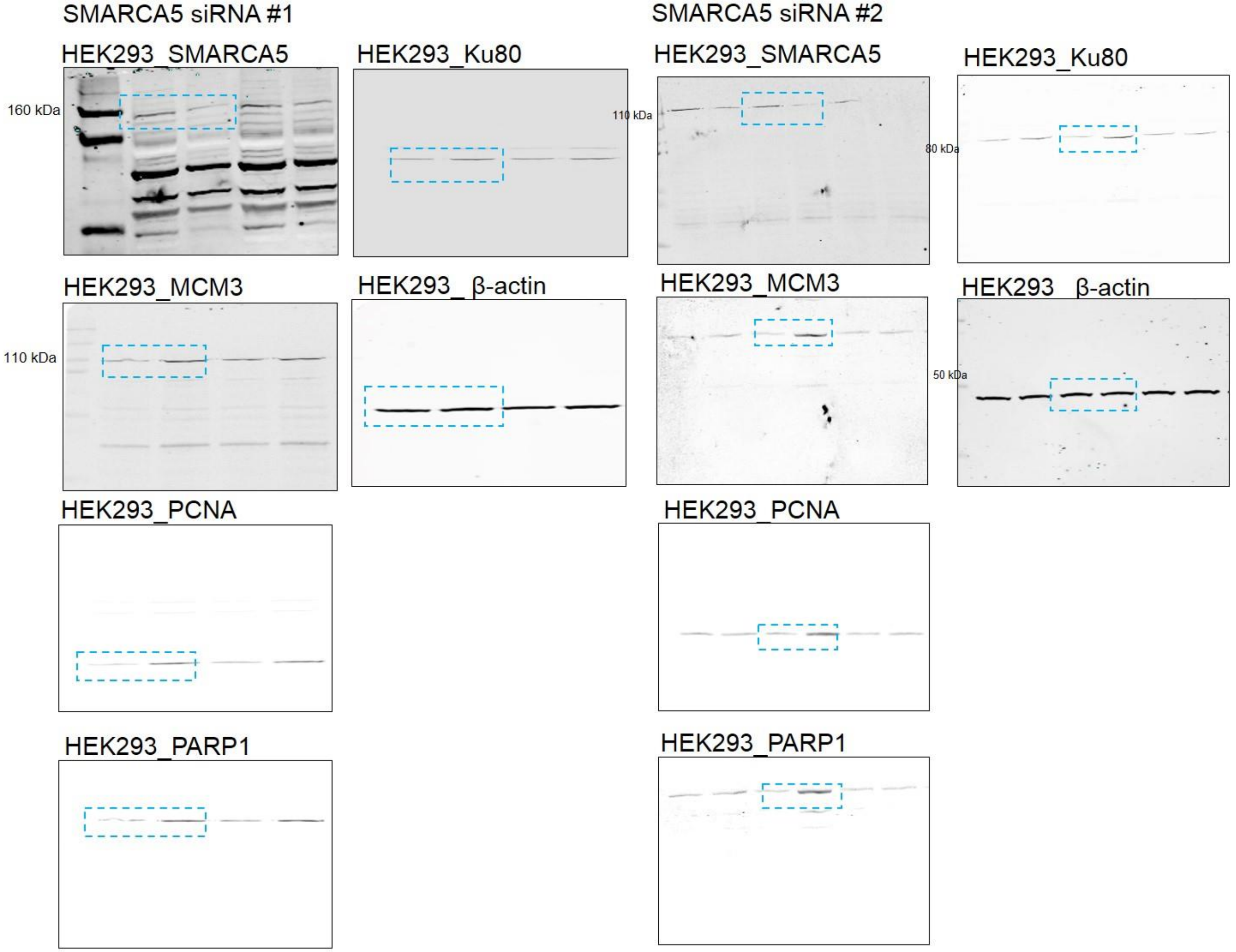

Supplementary Fig. 5b

Unprocessed blot-9

HPDE-1_pH2AX (Ser 139)

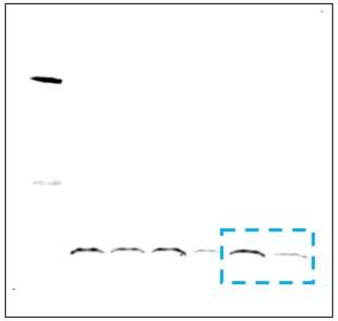

HPDE-1_H2AX

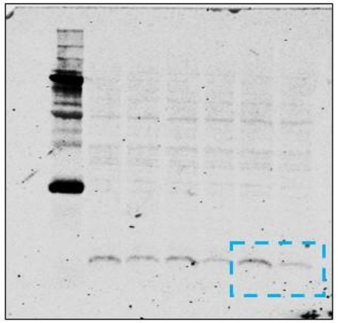

HPDE-1_β-actin

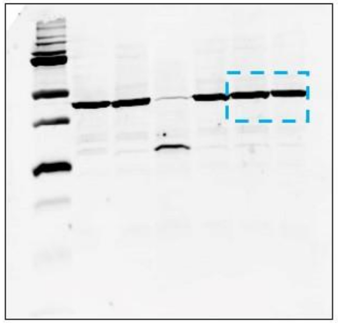

HEK293_pH2AX (Ser 139)

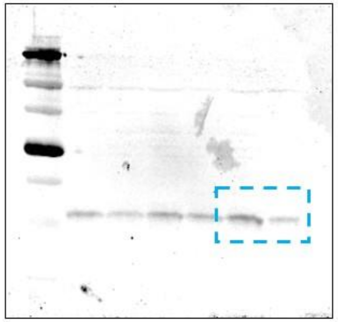

HEK293_H2AX

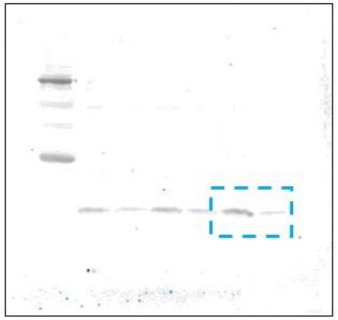

HEK293_β-actin

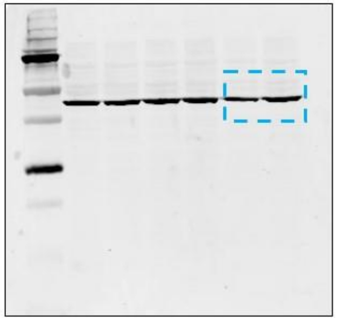

Unprocessed blot-10

Supplementary Fig. 5d

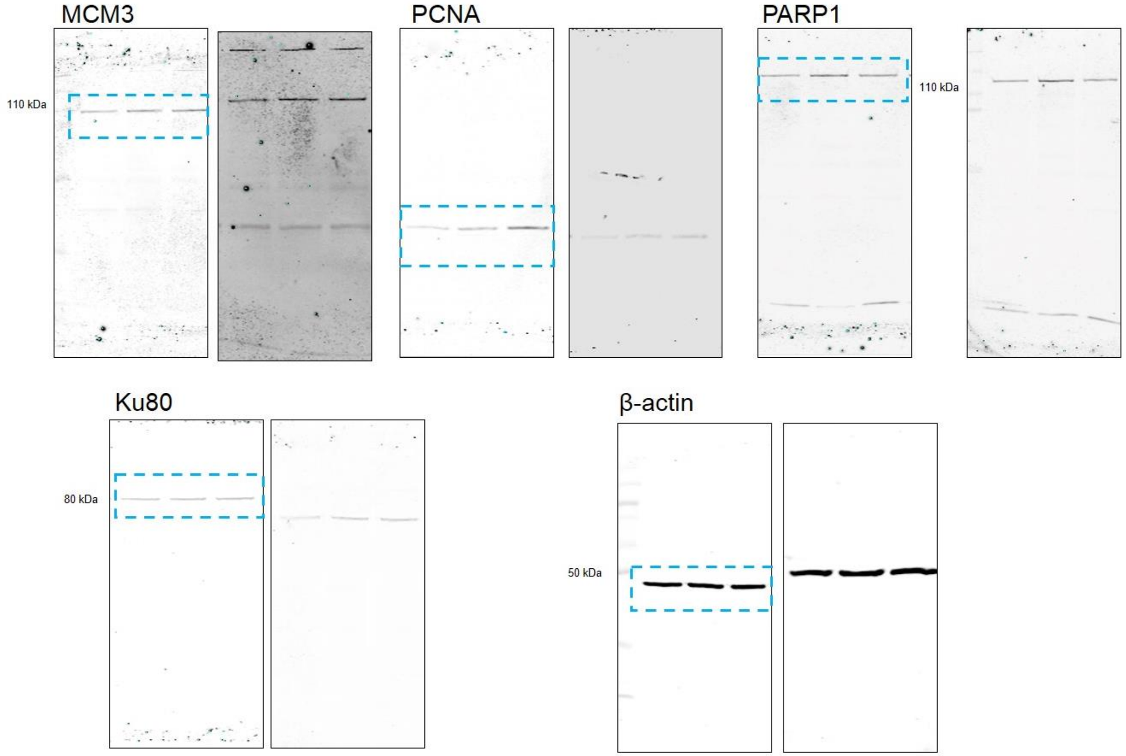

Supplement: Supplementary file 1 — Supplementary Information [file 42003_2020_1434_MOESM1_ESM.pdf]
