# Supplementary material for: Aberrant (pro)renin receptor expression induces genomic instability in pancreatic ductal adenocarcinoma through upregulation of SMARCA5/SNF2H
Source: Commun Biol. 2020 Nov 27;3:724. doi: 10.1038/s42003-020-01434-x (PMC7695732; doi:10.1038/s42003-020-01434-x)
Supplement: Supplementary file 3 — Description of Additional Supplementary Files [file 42003_2020_1434_MOESM3_ESM.pdf]

Description of Additional Supplementary Files

File Name: Supplementary Data 1
Description: The number of somatic mutations and SVs in either Mock-or (P)RR-expressing cells.

File Name: Supplementary Data 2
Description: The detailed information of somatic mutations in either Mock-or (P)RR-expressing cells.

File Name: Supplementary Data 3
Description: The detailed information of SVs in either Mock-or (P)RR-expressing cells.

File Name: Supplementary Data 4
Description: The detailed information of LC-MS/MS.

File Name: Supplementary Data 5
Description: The information of primers, antibodies, siRNAs and cell lines.
