# Supplementary material for: Aberrant (pro)renin receptor expression induces genomic instability in pancreatic ductal adenocarcinoma through upregulation of SMARCA5/SNF2H
Source: Commun Biol. 2020 Nov 27;3:724. doi: 10.1038/s42003-020-01434-x (PMC7695732; doi:10.1038/s42003-020-01434-x)
Supplement: Supplementary file 9 — Reporting Summary [file 42003_2020_1434_MOESM9_ESM.pdf]

nature research

Corresponding author(s): Akira Nishiyama

Last updated by author(s): Nov 12, 2020

Reporting Summary

Nature Research wishes to improve the reproducibility of the work that we publish. This form provides structure for consistency and transparency in reporting. For further information on Nature Research policies, see our Editorial Policies and the Editorial Policy Checklist.

Statistics

For all statistical analyses, confirm that the following items are present in the figure legend, table legend, main text, or Methods section.

n/a	Confirmed	
☐	☒	The exact sample size (*n*) for each experimental group/condition, given as a discrete number and unit of measurement
☐	☒	A statement on whether measurements were taken from distinct samples or whether the same sample was measured repeatedly
☐	☒	The statistical test(s) used AND whether they are one- or two-sided<br>*Only common tests should be described solely by name; describe more complex techniques in the Methods section.*
☒	☐	A description of all covariates tested
☒	☐	A description of any assumptions or corrections, such as tests of normality and adjustment for multiple comparisons
☐	☒	A full description of the statistical parameters including central tendency (e.g. means) or other basic estimates (e.g. regression coefficient) AND variation (e.g. standard deviation) or associated estimates of uncertainty (e.g. confidence intervals)
☐	☒	For null hypothesis testing, the test statistic (e.g. *F*, *t*, *r*) with confidence intervals, effect sizes, degrees of freedom and *P* value noted<br>*Give P values as exact values whenever suitable.*
☒	☐	For Bayesian analysis, information on the choice of priors and Markov chain Monte Carlo settings
☒	☐	For hierarchical and complex designs, identification of the appropriate level for tests and full reporting of outcomes
☒	☐	Estimates of effect sizes (e.g. Cohen's *d*, Pearson's *r*), indicating how they were calculated

Our web collection on statistics for biologists contains articles on many of the points above.

Software and code

Policy information about availability of computer code

Data collection	No software was used.
Data analysis	HiSeq Control Software v2.2.58 (Illumina; http://jp.support.illumina.com/sequencing/sequencing_instruments/hiseq_2500/downloads.html?langsel=/jp/), BWA (Li et al. 2009; http://bio-bwa.sourceforge.net/), SAMtools (Li et al. 2009; http://samtools.sourceforge.net/), RIKEN mutation calling with VAF for whole-genome sequencing (http://emu.src.riken.jp/MCV/), Xcalibur software (v4.0; Thermo Fisher Scientific; https://www.thermofisher.com/order/catalog/product/OPTON-30487), MASCOT (v2.6.1; Matrix Science; http://www.matrixscience.com/mascot_support_v2_6.html), Proteome Discoverer (v2.1.1.21; Thermo Fisher Scientific; https://www.thermofisher.com/order/catalog/product/OPTON-30795), Perseus (Tyanova et al.,2016; http://www.biochem.mpg.de/5111810/perseus), Ingenuity Pathway Analysis (Tomy Digital Biology; http://www.digital-biology.co.jp/allianced/products/ipa/), Image J (National Institutes of Health; https://imagej.nih.gov/ij/), OpenComet (Gyori et al, 2014; http://www.cometbio.org/), FlowJo (Tomy Digital Biology; http://www.digital-biology.co.jp/allianced/information/flowjo/), IBM SPSS Statistics 20 (IBM; https://www.ibm.com/software/jp/marketplace/spss/), R (The R Foundation for Statistical Computing Platform; https://www.r-project.org/).

For manuscripts utilizing custom algorithms or software that are central to the research but not yet described in published literature, software must be made available to editors and reviewers. We strongly encourage code deposition in a community repository (e.g. GitHub). See the Nature Research guidelines for submitting code & software for further information.

Data

Policy information about availability of data

All manuscripts must include a data availability statement. This statement should provide the following information, where applicable:

- Accession codes, unique identifiers, or web links for publicly available datasets
- A list of figures that have associated raw data
- A description of any restrictions on data availability

All the data produced in this study are stored at Department of Pharmacology, Faculty of Medicine, Kagawa University and are available from the corresponding author upon reasonable request.

Field-specific reporting

Please select the one below that is the best fit for your research. If you are not sure, read the appropriate sections before making your selection.

☒ Life sciences ☐ Behavioural & social sciences ☐ Ecological, evolutionary & environmental sciences

For a reference copy of the document with all sections, see nature.com/documents/nr-reporting-summary-flat.pdf

Life sciences study design

All studies must disclose on these points even when the disclosure is negative.

Sample size	Experimental group size was based on several previous studies, which is allowed us to perform the statistical analysis. For animals, we dedicated to the limited use .
Data exclusions	No data was excluded.
Replication	Where replication is shown in the manuscript, the reproducibility of experimental findings was verified.
Randomization	For animal studies, randomization was performed.
Blinding	Yes, this was done for DNA fibre and comet assays.

Reporting for specific materials, systems and methods

We require information from authors about some types of materials, experimental systems and methods used in many studies. Here, indicate whether each material, system or method listed is relevant to your study. If you are not sure if a list item applies to your research, read the appropriate section before selecting a response.

Materials & experimental systems

n/a	Involved in the study	
☐	☒	Antibodies
☐	☒	Eukaryotic cell lines
☒	☐	Palaeontology and archaeology
☐	☒	Animals and other organisms
☒	☐	Human research participants
☒	☐	Clinical data
☒	☐	Dual use research of concern

Methods

n/a	Involved in the study	
☒	☐	ChIP-seq
☐	☒	Flow cytometry
☒	☐	MRI-based neuroimaging

Antibodies

Antibodies used	The following antibodies were used in our study. ATP6AP2 (Sigma-Aldrich; SAB2702080; aa.146-350), ATP6AP2 (Hirose et al. 2009; aa.224-237), PCNA (Cell Signaling Technology;13110), PARP (Cell Signaling Technology; 9532), MCM3 (Cell Signaling Technology; 4003), Ku80 (Cell Signaling Technology; 2753), SP-1 (Cell Signaling Technology; 9389), Lamin B1 (Cell Signaling Technology; 12586), α-Tubulin (Cell Signaling Technology; 3873), β -actin (Sigma-Aldrich; A5441), phospho-Histone H2A.X Ser.139 (Cell Signaling Technology; 2577), Histone H2A.X (Cell Signaling Technology; 7631), phospho-p53 Ser.15 (Cell Signaling Technology; 9286), p53 (Cell Signaling Technology; 9282), Normal Rabbit IgG (Cell Signaling Technology; 2729), SNF2H (Abcam; ab3749), BrdU (Becton Dickinson; 347580), BrdU [BU1/75(ICR1)] (Abcam; ab6326), DYKDDDDK Tag (Thermo Fisher Scientific; MA1-91878), Alexa anti-mouse 488 (Thermo Fisher Scientific; A11001), Alexa-anti rat 594 (Thermo Fisher Scientific; A11007), Alexa anti-rabbit 488 (Thermo Fisher Scientific; A11034), IRDye 800 goat anti-rabbit IgG (LI-COR; 926-32221), IRDye 680 goat anti-mouse IgG (LI-COR; 926-32220).
Validation	Validation was based on information in the datasheets provided by manufactures.

Eukaryotic cell lines

Policy information about cell lines

Cell line source(s)	HPDE-1/E6E7 and HPDE-6/E6E7 cells were established by Furukawa et al. (1996) and Ouyang et al. (2000), respectively, which were gifted from Dr. Furukawa. PANC-1 and MiaPaCa-2 were from ATCC. Human T-cell leukemia 1301 was from ECACC. PK-1 was originally from IDAC. HEK293 was drawn from stocks at Department of Pharmacology, Faculty of Medicine, Kagawa University.
Authentication	The cell lines were not recently authenticated.
Mycoplasma contamination	All the cell lines used in this study have been tested for mycoplasma by MicoAlert (Lonza; BWLT07) and it is considered that cell lines used in this study are mycoplasma free.
Commonly misidentified lines (See ICLAC register)	No commonly misidentified lines were used in the study.

Animals and other organisms

Policy information about studies involving animals; ARRIVE guidelines recommended for reporting animal research

Laboratory animals	Laboratory animals used in this study were 5-week old male BALB/c immunodeficient mice (nu+/nu+; xenografts).
Wild animals	No wild animals were used in this study.
Field-collected samples	No field-collected samples were used in this study.
Ethics oversight	Approval was obtained from the Animal Experimentation Ethics Committee of Kagawa University.

Note that full information on the approval of the study protocol must also be provided in the manuscript.

Flow Cytometry

Plots

Confirm that:

- [x] The axis labels state the marker and fluorochrome used (e.g. CD4-FITC).
- [x] The axis scales are clearly visible. Include numbers along axes only for bottom left plot of group (a 'group' is an analysis of identical markers).
- [x] All plots are contour plots with outliers or pseudocolor plots.
- [x] A numerical value for number of cells or percentage (with statistics) is provided.

Methodology

Sample preparation	Detailed sample preparation is described in the Method section.
Instrument	MoFlo Astrios High-speed cell sorter
Software	Summit (v.6.0) and FlowJo (v.7.6.5/9.7/10)
Cell population abundance	No cell sorting was conducted.
Gating strategy	The axes showing FSC and SSC were used to discern single cells from doublet and multiple cells. The boundary between test cells and Human T-cell leukemia 1301 cells as an internal control was determined by fluorescence intensity of PI and FITC.

- [x] Tick this box to confirm that a figure exemplifying the gating strategy is provided in the Supplementary Information.
